# Effective high compression of ECG signals at low level distortion

**DOI:** 10.1038/s41598-019-40350-x

**Published:** 2019-03-14

**Authors:** Laura Rebollo-Neira

**Affiliations:** Mathematics Department, Aston University, B4 7ET Birmingham, UK

## Abstract

An effective method for compression of ECG signals, which falls within the transform lossy compression category, is proposed. The transformation is realized by a fast wavelet transform. The effectiveness of the approach, in relation to the simplicity and speed of its implementation, is a consequence of the efficient storage of the outputs of the algorithm which is realized in compressed Hierarchical Data Format. The compression performance is tested on the MIT-BIH Arrhythmia database producing compression results which largely improve upon recently reported benchmarks on the same database. For a distortion corresponding to a percentage root-mean-square difference (PRD) of 0.53, in mean value, the achieved average compression ratio is 23.17 with quality score of 43.93. For a mean value of PRD up to 1.71 the compression ratio increases up to 62.5. The compression of a 30 min record is realized in an average time of 0.14 s. The insignificant delay for the compression process, together with the high compression ratio achieved at low level distortion and the negligible time for the signal recovery, uphold the suitability of the technique for supporting distant clinical health care.

## Introduction

The electrocardiogram, frequently called ECG, is a routine diagnostic test to assess the electrical and muscular functions of the heart. A trained person looking at an ECG record can for instance interpret the rate and rhythm of heartbeats; estimate the size of the heart, the health of its muscles and its electrical systems; check for effects or side effects of medications on the heart, or check heart abnormalities caused by other health conditions. At the present time, ambulatory ECG monitoring serves to detect and characterize abnormal cardiac functions during long hours of ordinary daily activities. Thereby the validated diagnostic role of ECG recording has been extended beyond the bedside^1–3^.

The broad use of ECG records, in particular as a mean of supporting clinical health care from a distance, enhances the significance of dedicated techniques for compressing this type of data. Compression of ECG signals may be realized without any loss in the signal reconstruction, what is referred to as lossless compression, or allowing some distortion which does not change the clinical information of the data. The latter is called lossy compression. This procedure can enclose an ECG signal within a file significantly smaller than that containing the uncompressed record.

The literature concerning both lossless^4–8^ and lossy compression^9–15^ of ECG records is vast. It includes emerging methodologies based on compressed sensing^16–19^. This work focusses on lossy compression with good performance at low distortion recovery. Even if the approach falls within the standard transform compression category, it achieves stunning results. Fresh benchmarks on the MIT-BIH Arrhythmia database are produced for values of PRD as in recent publications^11,12,14,15^.

The transformation step applies a Discrete Wavelet Transform (DWT). It is recommended to use the fast Cohen-Daubechies-Feauveau 9/7 (CDF 9/7) DWT^20^, but other possibilities could also be applied. Techniques for ECG signal compression using a wavelet transform have been reported in numerous publications. For a review paper with extensive references see^21^. The main difference introduced by our proposal lies in the compression method. In particular in what we refer to as the Organization and Storage stage. One of the findings of this work is the appreciation that remarkable compression results are achievable even prescinding from the typical entropy coding step for saving the outputs of the algorithm. High compression is attained in straightforward manner by saving in the Hierarchical Data Format (HDF)^22^. More precisely, in the compressed HDF5 version which is supported by a number of commercial and non-commercial software platforms including MATLAB, Octave, Mathematica, and Python. HDF5 also implements a high-level Application Programming Interface (API) with C, C++, Fortran 90, and Java interfaces. As will be illustrated here, if implemented in software, adding to the algorithm an entropy coding process may improve compression further, but at expense of processing time. Either way, the compression results for distortion corresponding to mean PRD in the range [0.48, 1.71] are shown to significantly improve recently reported benchmarks^11,12,14,15^ on the MIT-BIH Arrhythmia database. For PRD < 0.4 the technique becomes less effective.

## Method

Before describing the method let’s introduce the notational convention. ${\mathbb{R}}$ is the set of real numbers. Bold face lower cases are used to represent one dimension arrays and standard mathematical fonts to indicate their components, e.g. ${\bf{c}}\in {{\mathbb{R}}}^{N}$ is an array of *N* real components *c*(*i*),*i* = 1, …, *N*, or equivalently **c** = (*c*(1), …, *c*(*N*)). Within the algorithms, operations on components will be indicated with a dot, e.g. **c**.^2^ = (*c*(1)^2^, …, *c*(*N*)^2^) and |**c**.| = (|*c*(1)|, …, |*c*(*N*)|). Moreover **t** = cumsum (|**c**.|^2^) is a vector of components $t(n)={\sum }_{i\mathrm{=1}}^{n}\,{|c(i)|}^{2},\,\,n=\mathrm{1,}\,\ldots ,\,N$.

The proposed compression algorithm consists of three distinctive steps.

1. *Approximation Step*. Applies a DWT to the signal keeping the largest coefficients to produce an approximation of the signal up to the target quality.
2. *Quantization Step*. Uses a scalar quantizer to convert the wavelet coefficients in multiples of integer numbers.
3. *Organization and Storage Step*. Organizes the outputs of steps (1) and (2) for economizing storage space.

At the Approximation Step a DWT is applied to convert the signal ${\bf{f}}\in {{\mathbb{R}}}^{N}$ into the vector ${\bf{w}}\in {{\mathbb{R}}}^{N}$ whose components are the wavelet coefficients (*w*(1), …, *w*(*N*)). For deciding on the number of nonzero coefficients to be involved in the approximation we consider two possibilities:

- The wavelet coefficients (*w*(1), …, *w*(*N*)) are sorted in ascending order of their absolute value (*w*(*γ*_1_), …, *w*(*γ*_*N*_)), with |*w*(*γ*_1_)|≤ ⋯ ≤|*w*(*γ*_*N*_)|. The cumulative sums $t(n)={\sum }_{i=1}^{k}\,{|w({\gamma }_{i})|}^{2},\,n=\mathrm{1,}\,\ldots ,\,N$ are calculated to find all the values *n* such that *t*(*n*) ≥ tol^2^. Let *k* + 1 be the smallest of these values. Then the indices *γ*_*i*_,*i* = *k* + 1,…*N* give the coefficients *w*(*γ*_*i*_), *i* = *k* + 1, …, *N* of largest absolute value. Algorithm 1 summarizes the procedure.
- After the quantization step the nonzero coefficients and their corresponding indices are gathered together.

**Algorithm 1:**
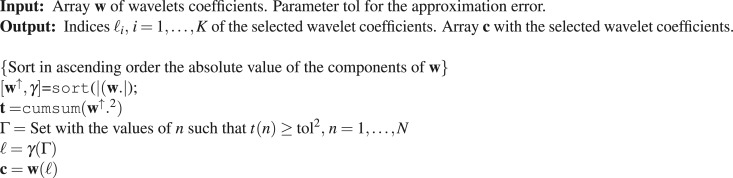
Selection of the largest wavelet coefficients Procedure $[{\bf{c}},\ell ]={\rm{SLWC}}({\bf{w}},\,{\rm{tol}})$.

At the Quantization Step the selected wavelet coefficients **c** = (*c*(1), …, *c*(*K*)), with *K* = *N* − *k* and *c*(*i* − *k*) = *w*(*γ*_*i*_), *i* = *k* + 1, …, *N*, are transformed into integers by a mid-tread uniform quantizer as follows:

1 $${c}^{{\rm{\Delta }}}(i)=\lfloor \frac{c(i)}{{\rm{\Delta }}}+\frac{1}{2}\rfloor ,\,i=\mathrm{1,}\,\ldots ,\,K.$$

where $\lfloor x\rfloor$ indicates the largest integer number smaller or equal to *x* and Δ is the quantization parameter. After quantization, the coefficients and indices are further reduced by the elimination of those coefficients which are mapped to zero by the quantizer. The above mentioned option (b) follows from this process. It comes into effect by skipping Algorithm 1. The signs of the coefficients are encoded separately using a binary alphabet (1 for + and 0 for −) in an array (*s*(1), …, *s*(*K*)).

Since the indices ${\ell }_{i},\,i=\mathrm{1,}\,\ldots ,\,K$ are large numbers, in order to store them in an effective manner at the Organization and Storage Step we proceed as follows. These indices are re-ordered in ascending order ${\ell }_{i}\to {\tilde{\ell }}_{i},\,i=\mathrm{1,}\,\ldots ,\,K$, which guarantees that ${\tilde{\ell }}_{i} < {\tilde{\ell }}_{i+1},\,i=\mathrm{1,}\,\ldots ,\,K$. This induces a re-order in the coefficients, ${{\bf{c}}}^{{\rm{\Delta }}}\to {\tilde{{\bf{c}}}}^{{\rm{\Delta }}}$ and in the corresponding signs ${\bf{s}}\to \tilde{{\bf{s}}}$. The re-ordered indices are stored as smaller positive numbers by taking differences between two consecutive values. Defining $\delta (i)={\tilde{\ell }}_{i}-{\tilde{\ell }}_{i-1},\,i=\mathrm{2,}\,\ldots ,\,K$ the array $\tilde{{\boldsymbol{\delta }}}=({\tilde{\ell }}_{1},\,\delta \mathrm{(2),}\,\ldots ,\,\delta (K))$ stores the indices ${\tilde{\ell }}_{1},\,\ldots ,\,{\tilde{\ell }}_{K}$ with unique recovery. The size of the signal, *N*, the quantization parameter Δ, and the arrays ${\tilde{{\bf{c}}}}^{{\rm{\Delta }}}$, $\tilde{{\bf{s}}}$, and $\tilde{{\boldsymbol{\delta }}}$ are saved in HDF5 format. The HDF5 library operates using a chunked storage mechanism. The data array is split into equally sized chunks each of which is stored separately in the file. Compression is applied to each individual chunk using gzip. The gzip method is based of on the DEFLATE algorithm, which is a combination of LZ77^23^ and Huffman coding^24^. Within MATLAB all this is implemented simply by using the function save to store the data.

Algorithm 2 outlines a pseudo code of the above described compression procedure.

**Algorithm 2:**
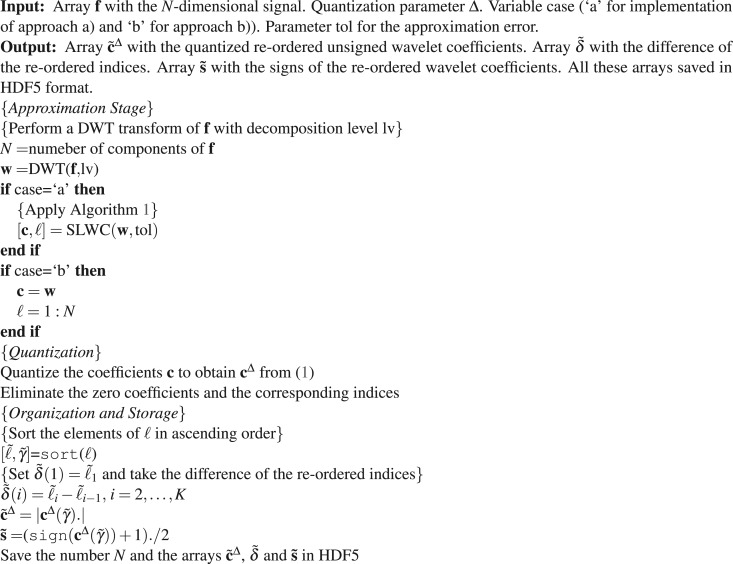
Compression Procedure.

The fast wavelet transform has computational complexity O(*N*). Thus, if the approach (a) is applied, the computational complexity of Algorithm 2 is dominated by the sort operation in Algorithm 1 with average computational complexity O(*NlogN*). Otherwise the complexity is just O(*N*), because the number *K* of indices of nonzero coefficients to be sorted is in general much less than *N*. Nevertheless, as will be shown in the Numerical Example III, in either case the compression of a 30 min record is achieved on a MATLAB platform in an average time less then 0.2 s. While compression performance can be improved further by adding an entropy coding step before saving the arrays, if implemented in software such a step slows the process.

When selecting the number of wavelet coefficients for the approximation by method a) the parameter tol is fixed as follows: Assuming that the target PRD before quantization is PRD_0_ we set ${\rm{tol}}={{\rm{PRD}}}_{{\rm{0}}}\Vert f\Vert /100$. The value of PRD_0_ is fixed as 70–80% of the required PRD. The quantization parameter is tuned to achieve the required PRD.

### Signal recovery

At the Decoding Stage the signal is recovered by the following steps.

- Read the number *N*, the quantization parameter Δ, and the arrays ${\tilde{{\bf{c}}}}^{{\rm{\Delta }}}$, $\tilde{{\boldsymbol{\delta }}}$, and $\tilde{{\bf{s}}}$ from the compressed file.
- Recover the magnitude of the coefficients from their quantized version as
  2 $${\tilde{{\bf{c}}}}^{{\rm{r}}}={\rm{\Delta }}{\tilde{{\bf{c}}}}^{{\rm{\Delta }}}.$$
- Recover the indices $\tilde{\ell }$ from the array $\tilde{{\boldsymbol{\delta }}}$ as: ${\tilde{\ell }}_{1}=\tilde{\delta }\mathrm{(1)}$ and ${\tilde{\ell }}_{i}=\tilde{\delta }(i)+\tilde{\delta }(i-\mathrm{1),}\,i=\mathrm{2,}\,\ldots ,\,K\mathrm{.}$
- Recover the signs of the the wavelet coefficients as ${\tilde{{\bf{s}}}}^{{\rm{r}}}=2\tilde{{\bf{s}}}-1$
- Complete the full array of wavelet coefficients as **w**^r^(*i*) = 0, *i* = 1, …, *N* and ${{\bf{w}}}^{{\rm{r}}}(\tilde{\ell })={\tilde{{\bf{s}}}}^{{\rm{r}}}\mathrm{.}{\tilde{{\bf{c}}}}^{{\rm{r}}}$
- Invert the wavelet transform to recover the approximated signal **f**^r^.

As shown in Tables 5–7, and the recovery process runs about 3 times faster than the compression procedure, which is already very fast.

## Results

We present here four numerical tests with different purposes. Except for the comparison in Test II, all the other tests use the full MIT-BIH Arrhythmia database^25^ which contains 48 ECG records. Each of these records consists of *N* = 650000 11-bit samples at a frequency of 360 Hz. The algorithms are implemented using MATLAB in a notebook Core i7 3520 M, 4GB RAM.

Since the compression performance of lossy compression has to be considered in relation to the quality of the recovered signals, we introduce at this point the measures to evaluate the results of the proposed procedure.

The quality of a recovered signal is assessed with respect to the PRD calculated as follows,

3 $${\rm{PRD}}=\frac{\Vert {\bf{f}}-{{\bf{f}}}^{{\rm{r}}}\Vert }{\Vert {\bf{f}}\Vert }\times 100 \% ,$$

where, **f** is the original signal, **f**^r^ is the signal reconstructed from the compressed file and $\Vert \cdot \Vert$ indicates the 2-norm. Since the PRD strongly depends on the baseline of the signal, the PRDN, as defined below, is also reported.

4 $${\rm{PRDN}}=\frac{\Vert {\bf{f}}-{{\bf{f}}}^{{\rm{r}}}\Vert }{\Vert {\bf{f}}-\overline{{\bf{f}}}\Vert }\times 100 \% ,$$

where, $\overline{{\bf{f}}}$ indicates the mean value of **f**.

When fixing a value of PRD, the compression performance is assessed by the Compression Ratio (CR) as given by

5 $${\rm{C}}{\rm{R}}=\frac{{\rm{S}}{\rm{i}}{\rm{z}}{\rm{e}}\,{\rm{o}}{\rm{f}}\,{\rm{t}}{\rm{h}}{\rm{e}}\,{\rm{u}}{\rm{n}}{\rm{c}}{\rm{o}}{\rm{m}}{\rm{p}}{\rm{r}}{\rm{e}}{\rm{s}}{\rm{s}}{\rm{e}}{\rm{d}}\,{\rm{f}}{\rm{i}}{\rm{l}}{\rm{e}}}{{\rm{S}}{\rm{i}}{\rm{z}}{\rm{e}}\,{\rm{o}}{\rm{f}}\,{\rm{t}}{\rm{h}}{\rm{e}}\,{\rm{c}}{\rm{o}}{\rm{m}}{\rm{p}}{\rm{r}}{\rm{e}}{\rm{s}}{\rm{s}}{\rm{e}}{\rm{d}}\,{\rm{f}}{\rm{i}}{\rm{l}}{\rm{e}}}.$$

The quality score (QS), reflecting the tradeoff between compression performance and reconstruction quality, is the ratio:

6 $${\rm{QS}}=\frac{{\rm{CR}}}{{\rm{PRD}}}.$$

Since the PRD is a global quantity, in order to detect possible local changes in the visual quality of the recovered signal, we define the local PRD as follows. Each signal is partitioned in *Q* segments **f**_*q*_, *q* = 1 …, *Q* of *L* samples. The local PRD with respect to every segment in the partition, which we indicate as prd(*q*), *q* = 1, … *Q*, is calculated as

7 $${\rm{prd}}(q)=\frac{\Vert {{\bf{f}}}_{q}-{{\bf{f}}}_{q}^{{\rm{r}}}\Vert }{\Vert {{\bf{f}}}_{q}\Vert }\times 100 \% ,$$

where ${{\bf{f}}}_{q}^{{\rm{r}}}$ is the recovered portion of the signal corresponding to the segment *q*. For each record the mean value prd ($\overline{{\rm{prd}}}$) and corresponding standard deviation (std) are calculated as

8 $$\overline{{\rm{prd}}}=\frac{1}{Q}\sum _{q=1}^{Q}\,{\rm{prd}}(q)$$

and

9 $${\rm{std}}=\sqrt{\frac{1}{Q-1}\sum _{q=1}^{Q}\,{({\rm{prd}}(q)-\overline{{\rm{prd}}})}^{2}}.$$

The mean value prd with respect to all the records in the database is a double average $\overline{\overline{{\rm{prd}}}}$.

When comparing two approaches on a database we reproduce the same mean value PRD. The quantification of the relative gain in CR of one particular approach, say approach 1, in relation to another, say approach 2, is given by the quantity:

$${\rm{Gain}}=\frac{{{\rm{CR}}}_{1}-{{\rm{CR}}}_{2}}{{{\rm{CR}}}_{2}}\times 100 \% .$$

The gain in QS has the equivalent definition.

### Numerical test I

We start the tests by implementing the proposed approach using wavelet transforms corresponding to different wavelet families at different levels of decomposition. The comparison between different wavelet transforms is realized using approach (b), because within this option each value of PRD is uniquely determined by the quantization parameter Δ. Thus, the difference in CR is only due to the particular wavelet basis and the concomitant decomposition level. Table 1 shows the average CR (indicated as CR_b_) and corresponding standard deviation (std) with respect to the whole data set and for three different values of PRD. For each PRD-value CR_b_ is obtained by means of the following wavelet basis: db5 (Daubechies) coif4 (Coiflets) sym4 (Symlets) and cdf97 (Cohen-Daubechies-Feauveau). Each basis is decomposed in three different levels (lv).

**Table 1:** Comparison of CRs for three values of PRD when the proposed approach is implemented using different wavelets at decomposition levels 3, 4, and 5.

Family	lv	Δ	PRD	CR	std	Δ	PRD	CR	std	Δ	PRD	CR	std
db5	3	47	0.65	24.03	5.44	36	0.53	20.25	4.54	29	0.45	**17.53**	3.95
	4	52	0.65	**25.12**	6.07	39	0.53	**20.75**	4.97	30	0.45	17.37	4.08
	5	53	0.65	23.31	5.51	39	0.53	19.22	4.48	30	0.45	15.49	3.84
coif4	3	47	0.65	24.28	5.59	36	0.53	20.47	4.56	29	0.45	17.76	3.98
	4	52	0.65	**25.56**	6.35	39	0.53	**21.15**	5.09	31	0.45	**18.10**	4.28
	5	53	0.65	23.73	5.75	39	0.53	19.61	4.59	31	0.45	16.39	4.17
	3	47	0.65	23.65	5.30	36	0.53	19.95	4.43	28	0.45	17.01	3.78
sym4	4	52	0.65	**25.13**	6.16	39	0.53	**20.76**	4.91	30	0.45	**17.41**.	4.10
	5	53	0.65	23.64	5.75	39	0.53	19.42	4.50	30	0.45	15.84	3.98
	3	47	0.65	24.66	5.39	36	0.53	20.94	4.66	28	0.45	17.80	3.93
cdf97	4	52	0.65	**26.59**	6.42	39	0.53	**22.16**	5.27	30	0.45	**18.57**	4.39
	5	53	0.65	24.98	6.06	39	0.53	20.69	4.83	30	0.45	16.75	4.20

As observed in Table 1, on the whole the best CR is achieved with the biorthogonal basis cdb97 for lv = 4. In what follows all the results are given using this basis for decomposition level lv = 4.

Next we produce the CR for every record in the database for a mean value PRD of 0.53.

Table 2 shows the results obtained by approach (a) where the CR and QS produced by this method are indicated as CR_a_ and QS_a_, respectively. The PRD values for each of the records listed in the first column of Table 2 are given in the forth columns of those tables. The second and third columns show the values of $\overline{{\rm{prd}}}$ and the corresponding std for each record. The CR is given in the fifth column and the corresponding QS in sixth column of the table. The mean value CR obtained by method (b) for the same mean value PRD = 0.53 is CR_b_ = 22.16.

**Table 2:** Compression results with approach a), cdf97 DWT, lv = 4, Δ = 35, and PRD_0_ = 0.4217, for the 48 records in the MIT-BIH Arrhythmia Database listed in the first column of the left and right parts of the table.

Rec	$\overline{{\rm{prd}}}$	std	PRD	CRa	QSa	PRDN
100	0.52	0.02	0.52	28.65	55.01	12.99
101	0.51	0.08	0.52	28.32	54.92	9.56
102	0.52	0.03	0.52	29.15	55.89	13.36
103	0.52	0.04	0.52	26.32	50.86	7.88
104	0.52	0.12	0.53	21.23	40.08	10.37
105	0.52	0.06	0.53	20.07	38.08	6.39
106	0.51	0.07	0.52	20.46	39.49	6.97
107	0.54	0.05	0.54	14.30	26.66	3.10
108	0.52	0.09	0.52	22.52	43.11	8.51
109	0.52	0.05	0.52	23.80	45.42	5.16
111	0.52	0.04	0.52	26.71	51.55	10.01
112	0.54	0.06	0.55	28.11	51.45	10.52
113	0.52	0.02	0.52	22.89	43.93	6.29
114	0.51	0.04	0.52	31.85	61.80	14.94
115	0.53	0.03	0.53	22.02	41.68	6.75
116	0.58	0.04	0.58	12.84	22.05	3.71
117	0.54	0.03	0.54	33.70	62.86	9.59
118	0.61	0.07	0.62	12.11	19.69	6.16
119	0.55	0.02	0.55	18.01	32.67	4.42
121	0.53	0.06	0.53	38.74	73.18	7.59
122	0.55	0.02	0.55	21.36	38.58	6.49
123	0.54	0.03	0.54	28.08	52.05	8.05
124	0.54	0.05	0.54	26.03	48.21	5.07
200	0.52	0.07	0.53	16.51	31.19	7.00
201	0.51	0.05	0.52	37.62	72.79	13.23
202	0.51	0.05	0.51	30.41	59.57	8.51
203	0.54	0.08	0.54	13.64	25.11	5.46
205	0.52	0.03	0.52	30.27	57.77	12.83
207	0.50	0.11	0.52	30.31	58.72	7.23
208	0.52	0.07	0.53	15.98	30.38	5.43
209	0.52	0.07	0.53	16.43	31.08	9.79
210	0.51	0.09	0.51	26.30	51.08	9.80
212	0.54	0.08	0.54	13.28	24.37	8.18
213	0.54	0.03	0.54	13.60	25.09	3.99
214	0.52	0.05	0.52	21.45	41.45	5.48
215	0.54	0.06	0.54	15.10	27.95	9.53
217	0.52	0.03	0.52	18.13	34.83	4.22
219	0.54	0.03	0.54	18.69	34.44	4.50
220	0.54	0.03	0.54	24.21	44.77	7.79
221	0.51	0.04	0.51	24.05	46.93	8.46
222	0.51	0.07	0.52	24.48	47.17	13.88
223	0.54	0.03	0.54	22.24	41.46	6.10
228	0.52	0.08	0.52	19.23	36.91	7.51
230	0.52	0.06	0.52	21.36	41.04	7.28
231	0.52	0.04	0.52	27.10	51.81	9.56
232	0.51	0.07	0.51	34.34	66.73	15.50
233	0.53	0.05	0.53	15.74	29.59	4.89
234	0.52	0.03	0.52	24.47	47.10	7.65
**mean**	**0.53**	**0.05**	**0.53**	**23.17**	**43.93**	**8.08**
**std**	**0.02**	**0.02**	**0.02**	**6.67**	**13.23**	**3.06**

Table 3 shows the variations of the CR_a_ with different values of the parameter PRD_0_ in method (a).

**Table 3:** Comparison of the CR achieving PRD = 0.53 with method a) of the proposed approach for different values of the parameter PRD_0_.

PRD_0_	0.212	0.265	0.318	0.371	0.424	0.477
CR_a_	22.16	22.19	22.42	22.88	23.24	19.50
Δ	39	39	39	37	32	21

### Numerical test II

Here comparisons are carried out with respect to results produced by the set partitioning in hierarchical threes algorithm (SPHIT) approach proposed in^26^. Thus for this test we use the data set described in that publication. It consists of 10-min long segments from records 100, 101, 102, 103, 107, 108, 109, 111, 115, 117, 118, and 111. As indicated in the footnote of^26^ at pp 853, the given values of PRD correspond to the subtraction of a baseline equal to 1024. This has generated confusion in the literature, as often the values of PRD in Tables III of^26^ are unfairly reproduced for comparison with values of PRD obtained without subtraction of the 1024 base line. The values of PRD with and without subtraction of that baseline, which are indicated as PRD_B_ and PRD respectively, are given in Table 4. As seen in this table, for the same approximation there is an enormous difference between the two metrics. A fair comparison with respect to the results in^26^ should either involve the figures in the second row of Table 4 or, as done in^26^, the fact that a 1024 base line has been subtracted should be specified.

**Table 4:** Comparison with the results of Table III in^26^.

PRD_B_	1.19	1.56	2.46	2.96	3.57	4.85	6.49
PRD	0.11	0.15	0.23	0.28	0.35	0.47	0.63
CR^26^	4	5	8	10	12	16	20
CR_b_	4.16	5.29	8.79	11.24	14.11	19.13	24.64
Gain %	4	6	10	12	18	20	23
${{\rm{CR}}}_{{\rm{b}}}^{{\rm{Huff}}}$	5.20	6.57	10.53	13.19	16.04	21.31	26.92
Gain %	30	31	32	32	34	33	35
Δ	3.87	5.45	8.79	14.53	20.10	33.10	51.50

The figures in the 3rd row of Table 4 correspond to the CRs in^26^. The 4th row shows the CRs resulting from method (b) of the proposed approach without entropy coding and the 5th row the results of adding a Huffman coding step before saving the compressed data in HDF5 format. The last two rows show the quantization parameters Δ which produce the required values of PRD_B_ and PRD.

### Numerical test III

This numerical test aims at comparing our results with recently reported benchmarks on the full MIT-BIH Arrhythmia database for mean value PRD in the rage [0.23, 1.71]. To the best of our knowledge the highest CRs reported so far for mean value PRD in the range [0.8, 1.30) are those in^12^, and in the range (1.30,1.71] those in^14^. For PRD < 0.8 the comparison is realized with the results in^11^, as shown in Table 7. Table 5 compares our results against the results in Table III of^12^ and Table 6 against Table 1 of^14^. In both cases we reproduce the identical mean value of PRD. The differences are in the values of CR and QS. All the Gains given in Table 5 are relative to the results in^12^ while those given in Tables 6 and 7 are relative to the results in^14^ and^11^, respectively.

**Table 5:** Comparison between the average performance of the proposed method and the method in^12^ for the same mean value of PRD.

PRD	1.71	1.47	1.18	1.05	0.91	0.80
CR^12^	38.46	33.85	28.21	25.64	22.27	18.00
CR_a_	**62.48**	**56.78**	**47.04**	**41.24**	**37.68**	**33.05**
Gain %	62	68	67	61	69	84
CR_b_	60.33	53.07	44.37	40.29	35.69	31.86
Gain %	57	57	57	57	60	77
QS^12^	29.08	29.38	30.01	30.51	30.36	29.46
QS_a_	**36.55**	**38.63**	**39.83**	**39.20**	**41.61**	**41.55**
Gain %	26	31	33	28	37	41
QS_b_	35.86	36.81	38.33	39.16	39.96	40.80
Gain %	23	25	27	28	31	38
**t**^c^ (s) a)	0.13	0.14	0.14	0.14	0.14	0.14
**t**^c^ (s) b)	0.10	0.11	0.11	0.11	0.11	0.11
**t**^r^ (s)	0.04	0.05	0.05	0.05	0.05	0.05
PRD_0_ a)	1.303	0.966	0.830	0.830	0.635	0.627
Δ a)	119	126	93	67	71	51
Δ b)	177	147	113	98	82	69

**Table 6:** Comparison between the average performance of the proposed method and the method in^14^ for the same mean value of PRD.

PRD	1.71	1.47	1.29	1.14	1.03	0.94
CR^14^	42.27	35.53	30.21	25.99	22.80	20.38
CR_a_	**62.48**	**56.78**	**49.60**	**45.75**	**41.00**	**38.52**
Gain %	48	60	64	76	80	89
CR_b_	60.33	53.07	48.04	42.94	39.76	36.52
Gain %	43	49	59	65	74	79
QS^14^	33.41	32.58	31.53	30.23	29.19	28.44
QS_a_	**36.55**	**38.63**	**38.43**	**40.28**	**39.72**	**41.03**
Gain%	9	18	21	33	36	44
QS_b_	35.86	36.81	38.00	38.50	39.26	39.77
Gain %	7	15	19	24	29	33
**t**^c^ a) (s)	0.14	0.14	0.14	0.14	0.14	0.14
**t**^c^ b) (s)	0.10	0.11	0.11	0.11	0.11	0.11
**t**^r^ (s)	0.05	0.05	0.05	0.05	0.05	0.05
PRD_0_ a)	1.303	0.966	0.971	0.750	0.804	0.690
Δ a)	119	126	91	96	68	69
Δ b)	177	147	126	108	96	85

**Table 7:** Comparison between the average compression performance of the proposed method and the method in^11^ for the same mean value of PRD.

PRD	1.31	1.02	0.67	0.48	0.31	0.23
CR^11^	17.34	14.68	11.30	9.28	6.22	5.19
CR_a_	**49.99**	**40.57**	**27.79**	**19.84**	**10.59**	**7.75**
Gain%	188	176	146	114	70	49
CR_b_	48.72	39.47	27.24	19.84	10.33	7.36
Gain%	181	169	141	114	66	42
QS_a_	38.12	39.81	41.46	42.71	34.96	34.48
QS_b_	37.69	39.30	41.88	42.71	34.40	33.16
**t**^c^ (s) a)	0.14	0.14	0.14	0.14	0.15	0.16
**t**^c^ (s) b)	0.11	0.11	0.11	0.11	0.13	0.14
**t**^r^ (s)	0.05	0.05	0.05	0.05	0.05	0.06
PRD_0_ a)	1.000	0.794	0.562	0.380	0.224	0.193
Δ a)	90	67	36	33	16	9
Δ b)	129	95	54	33	16	10

As already remarked, and fully discussed in^27^, when comparing results from different publications care should be taken to make sure that the comparison is actually on the identical database, without any difference in baseline. From the information given in the papers producing the results we are comparing with (the relation between the values of PRD and PRDN) we can be certain that we are working on the same database^25^, which is described in^28^.

The parameters for reproducing the required PRD with methods (a) and (b) are given in the last 3 rows of Tables 5–7. The previous 3 rows in each table give, in seconds, the average time to compress (**t**^c^) and recover (**t**^r^) a record. As can be observed, the compression times of approaches (a) and (b) are very similar. The given times were obtained as the average of 10 independent runs. Notice that the CR in these tables do not include the additional entropy coding step.

**Figure 1:**
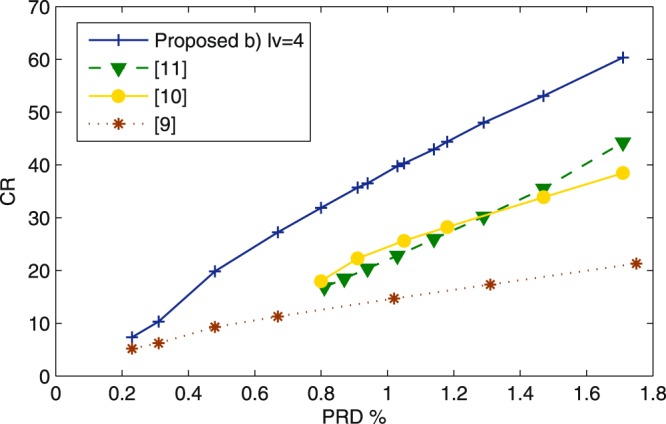
CR vs PRD corresponding to the proposed approach method (b) (blue line) and the approaches in^12^ (green line)^14^, (yellow line) and^11^ red line.

Figure 1 gives the plot of CR vs PRD for the approaches being compared in this section.

### Numerical test IV

Finally we would like to highlight the following two features of the proposed compression algorithm.

1. One of the distinctive features stems from the significance of saving the outputs of the algorithm directly in compressed HDF5 format. In order to highlight this, we compare the size of the file saved in this way against the size of the file obtained by applying a commonly used entropy coding process, Huffman coding, before saving the data in HDF5 format. The implementation of Huffman coding is realized, as in Table 4, by the off the shelf MATLAB functions Huff06 available on^29^. In Table 8 CR_a_ and CR_b_ indicate, as before, the CR obtained when the outputs of methods (a) and (b) are directly saved in HDF5 format. ${{\rm{CR}}}_{{\rm{a}}}^{{\rm{Huff}}}$ and ${{\rm{CR}}}_{{\rm{b}}}^{{\rm{Huff}}}$ indicate the CR when Huffman coding is applied on the outputs (a) and (b) before saving the data in HDF5 format. The rows right below the CRs give the corresponding compression times.

**Table 8:** Comparison of different storage methods. CR_a_ and CR_b_ are the CRs from approaches (a) and (b) when the outputs are saved directly in HFD5 format. ${{\rm{CR}}}_{{\rm{a}}}^{{\rm{Huff}}}$ and ${{\rm{CR}}}_{{\rm{b}}}^{{\rm{Huff}}}$ are the corresponding values when the Huffman codding step is applied before saving the data in HFD5 format.

PRD	1.0	0.9	0.8	0.7	0.6	0.5	0.4	0.3	0.2
CR_a_	40.51	37.12	33.09	29.70	25.50	22.00	16.80	10.33	6.64
${{\rm{CR}}}_{{\rm{a}}}^{{\rm{Huff}}}$	43.57	40.41	36.32	32.96	28.80	25.13	20.25	14.62	9.53
Gain %	8	9	10	11	13	14	20	42	43
**t**^c^ (s)	0.13	0.13	0.13	0.14	0.14	0.14	0.15	1.15	1.15
**t**^c^ (s)	4.3	4.5	5.0	5.4	5.5	6.2	8.3	10.22	15.4
Δ a)	71	64	50	45	35	30	24	15	8.5
PRD_0_ a)	0.750	0.675	0.640	0.550	0.484	0.400	0.300	0.230	0.150
CR_b_	38.64	35.41	31.86	28.53	24.93	21.03	16.21	10.07	6.56
${{\rm{CR}}}_{{\rm{b}}}^{{\rm{Huff}}}$	42.56	39.20	35.65	32.10	28.37	24.32	19.60	14.32	9.40
Gain %	10	11	12	13	14	16	21	42	43
**t**^c^ (s)	0.11	0.11	0.11	0.11	0.11	0.11	0.12	0.12	0.12
**t**^c^ (s)	4.1	4.0	4.5	5.2	5.4	6.7	8.1	10.7	15.5
Δ b)	92	81	69	58	47	36	25	15.5	8.5
CR_RL_	26.63	24.41	22.42	20.34	18.14	15,68	12.50	8.32	5.61
${{\rm{CR}}}_{{\rm{RL}}}^{{\rm{Huff}}}$	35.06	31.78	28.80	25.93	22.91	19.66	15.85	11.63	7.82
Gain %	32	30	28	27	26	25	26	40	40
**t**^c^ (s)	0.12	0.12	0.12	0.13	0.13	0.13	0.13	0.14	0.16
**t**^c^ (s)	4.5	4.9	6.2	6.4	7.1	7.4	9.0	12.8	19.5

CR_RL_ gives the CR if the outputs of method (b) are stored using the RL algorithm and the arrays are saved in HFD5 format. ${{\rm{CR}}}_{{\rm{RL}}}^{{\rm{Huff}}}$ is the corresponding CR if Huffman codding is applied before saving the arrays in HFD5 format.
2. The other distinctive feature of the method is the significance of the proposed Organization and Storage step. In order to illustrate this, we compare the results obtained by method (b) with those obtained using the conventional Run-Length (RL) algorithm^30^ instead of storing the indices of nonzero coefficients as proposed in this work. The CR corresponding to RL in HDF5 format is indicated in Table 8 as CR_RL_. When Huffman coding is applied on RL before saving the outputs in compressed HDF5 format, the CR is indicated as ${{\rm{CR}}}_{{\rm{RL}}}^{{\rm{Huff}}}$.

## Discussion

We notice that, while the results in Table 1 show some differences in CR when different wavelets are used for the DWT, it is clear from the table that the selection of the wavelet family is not the crucial factor for the success of the technique. The same is true for the decomposition level. That said, since the best results correspond to the cdf97 family at decomposition level 4, we have realized the other numerical tests with that wavelet basis.

**Figure 2:**
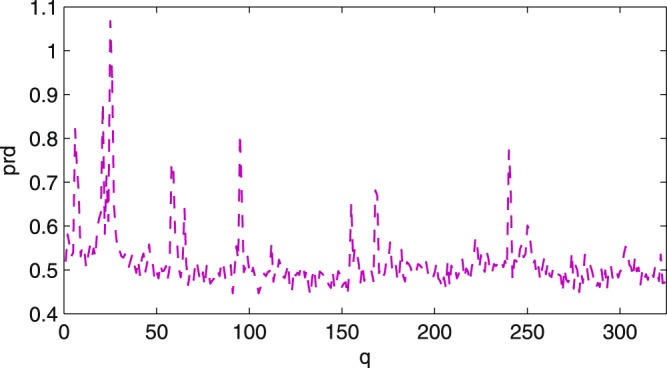
Values of prd for the *Q* = 325 segments of length *L* = 2000 in record 101.

We chose to produce full results for a mean value PRD of 0.53 (c.f. Table 2) as this value represents a good compromise between compression performance and high visual similitude of the recovered signal and the raw data. Indeed, in^15^ the quality of the recovered signals giving rise to a mean value PRD of 0.53 is illustrated in relation to the high performance of automatic QRS complex detection. However, the compression ratio of their method is low. For the same mean value of PRD our CR is 5 times larger: 4.5^15^ vs 23.17 (Table 2). As observed in Table 2 the mean value of the local quantity prd is equivalent to the global value (PRD). Nevertheless the prd may differ for some of the segments in a record. Figure 2 plots the prd for record 101 partitioned into *Q* = 325 segments of length *L* = 2000 sample points. Notice that there are a few segments corresponding to significantly larger values of prd than the others. Accordingly, with the aim of demonstrating the visual quality of the recovered signals, for each signal in the database we detect the segment ${q}^{\ast }$ of maximum distortion with respect to the prd as

10 $${q}^{\ast }=\mathop{{\rm{\arg }}\,{\rm{\max }}}\limits_{q\,=\,\mathrm{1,}\,\ldots ,\,Q}{\rm{prd}}(q\mathrm{).}$$

**Figure 3:**
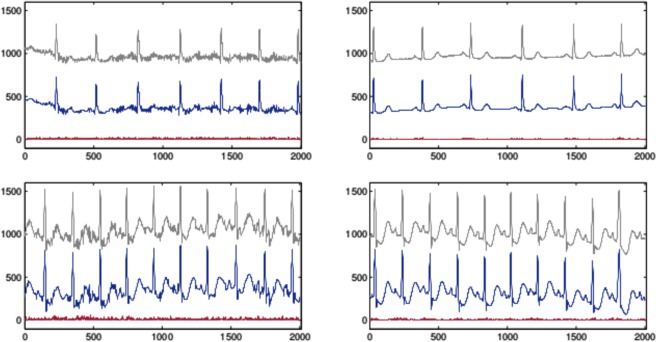
The upper waveforms in all the graphs are the raw data. The lower waveforms are the corresponding approximations which have been shifted down for visual convenience. The bottom lines represent the absolute value of the difference between the raw data and the approximation. The top left graph corresponds to segment 25 in record 101 and the right one corresponds to segment 120 in the same record. The bottom graphs have the same description as the top graphs but for record 213 and segment 175 (left) and 51 (right).

The left graphs of Fig. 3 correspond to the segments of maximum prd with respect to all the records in the database and segments of length *L* = 2000. These are: the segment 25 of records 101, when applying the approximation approach (a) (top graph), and segment 175 of record 213 for approach (b) (bottom graph). The upper waveforms in all the graphs are the raw ECG data. The lower waveforms are the corresponding approximations which have been shifted down for visual convenience. The bottom lines in all the graphs represent the absolute value of the difference between the raw data and their corresponding approximation. The right graphs of Fig. 3 have the same description as the left ones but the segments correspond to values of prd close to the mean value prd for the corresponding record.

It is worth commenting that the difference in the results between approaches (a) and (b) is consequence of the fact that the concomitant parameters are set to approximate the whole database at a fixed mean value PRD. In that sense, approach (a) provides us with some flexibility (there are two parameters to be fixed to match the required PRD) whereas for approach (b) the only parameter (Δ) is completely determined by the required PRD. As observed in Table 3, when setting the parameter PRD_0_ much smaller than the target PRD the approximation is only influenced by the quantization parameter Δ and methods (a) and (b) coincide. Contrarily, when setting the PRD_0_ too close to the target PRD the quantization parameter needs to be significantly reduced, which affects the compression results. For a target PRD≥0.4 we recommend to set PRD_0_ as 70–80% of the required PRD.

For values of PRD < 0.4 the storage approach is not as effective as for larger values of PRD. This is noticeable in both Tables 4 and 8. Another feature that appears for PRD < 0.4 is that applying the entropy coding step, before saving the data in compressed HDF5 format, improves the CR much more than for larger values of PRD. This is because for PRD < 0.4 the approximation fits noise and small details, for which components in higher wavelet bands are required. Contrarily, for larger values of PRD the adopted uniform quantization keeps wavelet coefficients in the first bands. As a result, through the proposed technique the location of the nonzero wavelet coefficients is encoded in an array which contains mainly a long stream of ones. For small values of PRD the array’s length increases to include different numbers. This is why the addition of an entropy coding step, such as Huffman coding which assigns smaller bits to the most frequent symbols, becomes more important. In any case, if the outputs are saved in HDF5 format, adding the Huffman coding step is beneficial. Nonetheless, since when implemented in software the improvement comes at expense of computational time, for PRD > 0.4 this step can be avoided and the CR is still very high.

Comparisons with the conventional RL algorithm, in Table 8, enhances the suitability of the proposal for storing the location of nonzero coefficients. A similar storage strategy has been successfully used with other approximation techniques for compression of melodic music^31^ and X-Ray medical images^32^. In this case the strategy is even more efficient, because the approximation is realized using a basis and on the whole signal, which intensifies the efficiency of the storage approach.

## Conclusions

An effective and efficient method for compressing ECG signals has been proposed. The proposal was tested on the MIT-BIH Arrhythmia database, which gave rise to benchmarks improving upon recently reported results. The main feature of the method is its simplicity and the fact that for values of PRD > 0.4 a dedicated entropy coding to save the outputs can be avoided by saving the outputs of the algorithm in compressed HDF5. This solution involves a time delay which is practically negligible in relation to the signal length: 0.14 s for compressing a 30 min record. Two approaches for reducing wavelet coefficients have been considered. Approach (b) arises from switching off in approach (a) the selection of the largest wavelet coefficients before quantization. It was shown that, when approximating a whole database to obtain a fixed mean value of PRD, approach (a) may render a higher mean vale of CR when the target PRD is greater the 0.4.

The role of the proposed Organization and Store strategy was highlighted by comparison with the conventional Run Length algorithm. Whilst the latter produces smaller CRs, the results are still good in comparison with previously reported benchmarks. This outcome leads to conclude that, using the a wavelet transform on the whole signal, uniform quantization for all the wavelet bands works well in the design of a codec for lossy compression of ECG signals.

**Note:** The MATLAB codes for implementing the whole approach have been made available on a dedicated website^29,33^.

## Data Availability

The data used in this paper are available on https://physionet.org/physiobank/database/mitdb/ We have also placed the data, together with the software for implementing the proposed approach, on http://www.nonlinear-approx.info/examples/node012.htm.
